# Unidigital clubbing in a patient with ulnar neuropathy due to Hansen's disease

**DOI:** 10.1055/s-0042-1760085

**Published:** 2022-12-29

**Authors:** Victor Evangelista, Sofia Abreu Mermelstein, Marcia Jardim

**Affiliations:** 1 Universidade Federal do Rio de Janeiro, Hospital Universitário Clementino Fraga Filho, Serviço de Neurologia, Departamento de Clínica Médica, Rio de Janeiro RJ, Brazil.; 2 Universidade Estadual do Rio de Janeiro, Hospital Universitário Pedro Ernesto, Departamento de Neurologia, Rio de Janeiro RJ, Brazil.

A 37-year-old woman presented with a 5-year history of skin lesions with multiple erythematous papules, enlarged nerves on palpation, and a right ulnar neuropathy, characterized by weakness and wasting of intrinsic hand muscles and numbness in the fifth and fourth fingers of the right hand, with unidigital clubbing. This is an unusual sign, previously reported in finger trauma, median nerve neuropathy, brachial plexopathy, sarcoidosis, and vascular conditions such as aortic aneurysms. To our knowledge, the present is the first report on ulnar neuropathy secondary to leprosy, confirmed by skin biopsy, and serves as a clue to the diagnosis of mononeuropathies. 1

**Figure 1:**
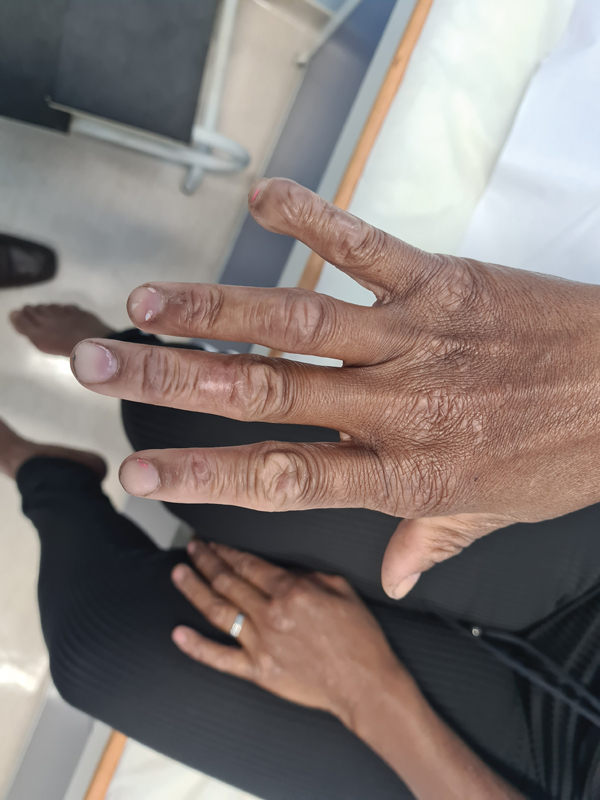
Patient's hand, showing unidigital clubbing of the right hand's fifth finger and wasting of the interosseous muscle as well as the fifth finger abduction sign (Wartenberg sign). 2
